# Enzymatic Degradation of Star Poly(*ε*-Caprolactone) with Different Central Units

**DOI:** 10.3390/polym10111266

**Published:** 2018-11-14

**Authors:** Catherine J. Blackwell, Karolina Haernvall, Georg M. Guebitz, Michael Groombridge, Denis Gonzales, Ezat Khosravi

**Affiliations:** 1 Department of Chemistry, Durham University, Durham DH1 3LE, UK; c.j.blackwell@durham.ac.uk (C.J.B.); ezat.khosravi@durham.ac.uk (E.K.); 2 Austrian Centre of Industrial Biotechnology GmbH, Konrad Lorenz Strasse 20, 3430 Tulln an der Donau, Austria; karolina.haernvall@gmail.com; 3 Institute for Environmental Biotechnology, University of Natural Resources and Life Sciences, Konrad Lorenz Strasse 20, 3430 Tulln an der Donau, Austria; 4 Procter & Gamble, Cobalt 12A, Silver Fox Way, Cobalt Business Park, Newcastle upon Tyne NE27 0QW, UK; groombridge.m@pg.com (M.G.); Gonzales.d@pg.com (D.G.)

**Keywords:** polycaprolactone, enzyme, biodegradation

## Abstract

Four-arm star poly(ε-caprolactone) with a central poly(ethylene glycol) PEG unit bridged with 2,2-*bis*(methyl) propionic acid, (PCL)_2_-*b*-PEG-*b*-(PCL)_2_, and six-arm star PCL homopolymer with a central dipentaerythritol units were hydrolysed using a lipase from *Pseudomonas cepacia* and the *Thermobifida cellulosilytica* cutinase Thc_Cut1. For comparative analysis, Y-shaped copolymers containing methylated PEG bridged with bisMPA, MePEG-(PCL)_2_, and linear triblock copolymers PCL-*b*-PEG-*b*-PCL were also subjected to enzymatic hydrolysis. The hydrophilic nature of the polymers was determined using contact angle analysis, showing that a higher PEG content exhibited a lower contact angle and higher surface wettability. Enzymatic hydrolysis was monitored by % mass loss, scanning electron microscopy (SEM), and differential scanning calorimetry (DSC). A higher rate of mass loss was found for lipase catalysed hydrolysis of those polymers with the highest PEG content, leading to significant surface erosion and increase in crystallinity within the first two days. Liquid chromatography (LC) and size exclusion chromatography (SEC) of samples incubated with the cutinase showed a significant decrease in molecular weight, increase in dispersity, and release of *ε*-CL monomer units after 6 h of incubation.

## 1. Introduction

Poly(*ε*-caprolactone) (PCL) has been shown to be non-toxic, biocompatible, and biodegradable [1,2,3]. It has received significant attention in applications for biomedical use, due to its good mechanical properties and tuneable degradation rate. It can be synthesised by the facile ring-opening polymerisation (ROP) of ε-caprolactone (*ε*-CL) using a nucleophilic initiator such as hydroxyl groups and catalysed by FDA-approved tin (II) ethyl hexanoate (SnOct_2_) [4,5]. Hydrophilic poly(ethylene glycol) (PEG) has been recognised as a biocompatible water soluble polymer with important biological or pharmaceutical/biomedical applications [6].

PEG has been copolymerised with hydrophobic aliphatic polyesters such as PCL and poly(lactide-*co*-glycolide) (PLGA) to increase the hydrophilic nature and therefore increase the rate of abiotic hydrolysis of the hydrophobic polyester block [7]. However, some studies have shown that the enzymatic degradation of PCL was not affected by the incorporation of a hydrophilic PEG unit [8,9].

The complete degradation of PCL in the presence of *Pseudomonas* lipase has been reported to occur within 4 days [10], whereas hydrolytic degradation in the absence of enzymes takes several years [11]. The enzymatic degradation of PCL has been extensively investigated using lipase [12,13,14]. It has been reported that only three kinds of lipases effectively accelerate the degradation of PCL which are obtained from fungi or bacteria, namely from *Rhizopus delemer* [15], *Rhizopus arrhizus* [16], and from *Pseudomonas* PS [13,17]. Different mechanisms of PCL degradation have been proposed; a surface erosion mechanism during enzymatic degradation, and a bulk erosion mechanism in hydrolytic degradation_ENREF_4 [18].

The synthesis and hydrolytic degradation of star copolymers consisting of a central PEG moiety with 2, 4, and 8 poly(lactic-*co*-glycolic acid) (PLGA) arms have been reported [7]. These star copolymers were degraded in PBS solution (pH = 7.4) at 37 °C and an increased degradation rate with an increased arm number or with decreased arm length was found. On the other hand, it has been reported that enzymatic degradation of PCL occurs though random chain scission of the polymer backbone, irrespective of chain length [19].

Enzymatic degradation of PCL has been shown to be dependent on crystallinity as the degree of crystallinity initially increases during degradation, indicating amorphous regions are primarily degraded [20,21]. Furthermore, it has been proposed that enzymatic degradation of PCL primarily occurs at the chain-ends, chain-folds, and at the edge of crystals, where chain mobility is higher [22]. Generally, PCL copolymers show a decreased crystallinity in comparison to PCL homopolymers therefore, which would be expected to increase the rate of enzymatic degradation.

Inglis et al. identified an extracellular cutinase (CutA) from *Pseudomonas pseudoalcaligenes* from a mixed-plant compost based on a polycaprolactone assay [23]. Murphy et al. determined that microbial cutinase production was induced by PCL. They also identified a PCL depolymerase from *Fusarium solani* which was later classified as cutinase [24]. In addition, Nishida and Tokiwa have shown that fungal phytopathogens can degrade PCL and indicated that their cutinases may act as PCL depolymerases [25].

In this study, we report on the enzymatic degradation of a series of star copolymers containing a central hydrophilic PEG moiety and four hydrophobic PCL arms, in comparison to linear and Y-shaped PCL-PEG copolymers. Enzymatic degradation of these polymers was investigated using a *Pseudomonas cepacia* lipase and the *Thermobifida cellulosilytica* cutinase (Thc_Cut1). The degradation of PCL polymers using cutinases has not yet been systematically investigated despite being shown to effectively degrade a variety of synthetic polyesters [26,27,28]. To the best of our knowledge, this is the only example of the enzymatic degradation of star copolymers containing a central PEG moiety and four PCL arms bridged with a *bis*MPA linkage.

## 2. Materials and Methods

### 2.1. Materials

All polymers were synthesised as in the literature [29,30,31,32]. Phosphate buffer salts (pH = 7.4) and *Pseudomonas cepacia* lipase, tetrahydrofuran, butylated hydroxytoluene, 4-nitrophenyl butyrate, and *ε*-Caprolactone were purchased from Sigma Aldrich (St. Louis, MI, USA) and used as received. 6-Hydroxyhexanoic acid was purchased from Alfa Aesar (Haverhill, MA, USA) used as received. Carrez reagent I and Carrez reagent II were prepared by dissolving K_4_[Fe(CN)_6_]·3H_2_O (5.325 g) and ZnSO_4_*7H_2_O (14.400 g) in MQ water (50 mL), respectively.

### 2.2. Measurements

Scanning Electron Microscopy (SEM) was carried out using Hitachi SU-70 FEG (Hitachi, Tokyo, Japan). The films were first sputter-coated in gold to provide good conductivity of the electron beam. A voltage of 25 kV and a probe current of 90 μA was used.

Differential Scanning Calorimetry (DSC) was carried out on a TA Instruments DSC Q1000 (TA Instruments, New Castle, DE, USA) over a temperature range of −80 to 150 °C at a rate of 10 °C min^−1^. The heating scans of the samples before and after enzymatic degradation with lipase were recorded. The enthalpy of fusion (Δ*H*_m_) was calculated from the heating scans and degree of crystallinity (%*χ*_c_) calculated from the cooling scans. Three repeat scans were taken for every sample.

Contact angle measurements were carried out on FTA200 equipped with a halogen bulb and a S1640 monochrome camera (First Ten Angstroms, Inc., Portsmouth, VA, USA). The de-ionised water droplet was automatically delivered to the polymer film using a syringe with a blunt 27 gauge needle. The camera recorded 750 images of the droplet and film over a period of 30 s. The contact angle (*θ*) was calculated by the average of the left and right angles. The reported contact angle measurements are averages of five repeat measurements.

Size Exclusion Chromatography (SEC) was carried out at 30 °C on an Agilent Technologies HPLC System (Agilent Technologies 1260 Infinity, Santa Clara, CA, USA) connected to a Guard column H_HR_-H (5 μm, 4 cm length, 6 mm ID) and a TSKgel GMH_HR_-N (5 μm, 30.0 cm length, 7.8 mm ID) liquid chromatography column (Tosoh Bioscience, Tokyo, Japan) using THF (250 ppm BHT as inhibitor) as eluent at a flow rate of 1 mL min^−1^. An Agilent Technologies G1362A refractive index detector was employed and kept at a temperature of 30 °C. The molecular weights of the polymers were calculated relative to polystyrene standards (250–2,000,000 Da).

Liquid Chromatography (LC) was carried out on a Hewlett Packard series 1100 system, equipped with an ion exclusion column ION 300 (Transgenomic Organic, Omaha, NE, USA) heated to 45 °C and a refractive index detector (Agilent 1100, Santa Clara, CA, USA). The mobile phase consisted of a 5 mM H_2_SO_4_ solution with a flow rate of 0.325 mL min^−1^.

### 2.3. Expression and Purification of Cutinase

The *Thermobifida cellulosilytica* cutinase (Thc_Cut1) was expressed in *E. coli* BL21-Gold(DE3) cells as previously reported [33]. An overnight culture of LB-medium (50 mL) supplemented with kanamycin (40 μg mL^−1^) were inoculated with freshly transformed *E. coli* BL21-Gold(DE3) cells and incubated on a rotary shaker at 37 °C and 130 rpm. The overnight culture was used to inoculate the main culture (400 mL) containing the same medium. The main culture was incubated on a rotary shaker at 37 °C and 140 rpm until an optical density (600 nm) of 0.6 were reached. Thereafter, the culture was induced with isothiopropyl-*β*-d-galactoside (IPTG) at a final concentration of 0.05 mM and incubated at 20 °C and 140 rpm for 21 h. Centrifugation was used for harvesting the cells (4500 g, 4 °C, 20 min, Sorvall RC-5B Refrigerated Superspeed Centrifuge, Du Pont Instruments, Wilmington, DE, USA). The supernatant was discarded and the pellet from 400 mL cell culture was re-suspended in lysis buffer (20 mM NaH_2_PO_4_, 500 mM NaCl and 10 mM imidazol, pH 7.4). The cells were disrupted by sonication (RANSON Ultrasonics cell disruptor, Danbury, CT, USA) for 6 min with a cycle of 1 s sonification and 4 s pause with an amplitude if 60%. The sonicated cells were centrifuged (Sorvall RC-5B Refrigerated Superspeed Centrifuge, Du Pont Instruments, Miami, FL, USA) for 60 min at 18,000 rpm at 4 °C to remove cell fragments prior enzyme purification. The supernatant was used for further purification. To enable purification a 6×His peptide was C-terminally fused over an Ala-Leu-Glu linker sequence to the cutinase. The cutinase was purified by immobilized metal ion affinity chromatography (IMAC) using HisTrap FF 5 mL columns coupled with ÄKTA purifier 900 (GE Healthcare, Little Chalfont, UK). The column was equilibrated with the lysis buffer and 30 mL cell free extract were loaded on the column. The run was performed with buffer (500 mM NaCl, 20 mM NaH_2_PO_4_ pH 7.4) and the enzyme was eluted by increasing the imidazole from 10 to 500 mM for 25 min (10 CV). The enzyme containing fractions were pooled and centrifuged with Vivaspin 20 column with a molecular weight cut-off of 10,000 Da (Sartorius AG, Göttingen, Germany) for concentration. The elution buffer was exchanged with 100 mM TRIS-HCl pH 7.0 by the use of PD-10 desalting columns (Amersham Biosciences, Little Chalfont, UK).

Bradford-based Bio-99 Rad Protein Assay (Bio-Rad Laboratories GmbH, Munich, Germany) with bovine serum albumin (BSA) as standard was used to determine the protein concentration of the purified enzymes. The protein assay was performed according to the manufacturers’ instruction. SDS-PAGE analysis was performed corresponding to Laemmli proteins were stained with Coomassie Brilliant Blue R-250 [34].

The cutinase production in *E. coli* BL21-Gold (DE3) resulted in strong protein bands below 30 kDa as determined by SDS-PAGE analysis of the soluble and insoluble cell fractions which corresponds well to the calculated mass of 29.4 kDa of Thc_Cut1. The cutinase were expressed intracellularly in the soluble and active form. Purification over 6×His-Tag resulted in high purity, where 10–15 mg purified enzyme was obtained from 100 mL cell culture.

### 2.4. Cutinase Enzyme Activity

The esterase activity of the enzymes was measured with a photometric esterase assay based on the soluble substrate *p*-nitrophenyl substrates as previously reported by Ribitsch et al [33]. Briefly, the soluble *p*-nitrophenyl substrates were prepared in a 50 mM potassium phosphate (pH 7.0) buffer solution. The activity measurements were performed in 200 μL of the buffer solution and started by addition of 20 μL of enzyme solution. A blank reaction was prepared in which 20 μL buffer were added without enzyme. The increased absorbance was continuously measured for 5 min at 405 nm on a plate reader (Tecan infinite M200, Tecan Austria GmbH, Groedig, Austria) at 25 °C. The hydrolysis of 4-nitrophenyl butyrate to *p*-nitrophenol leads to an absorbance increase at 405 nm (*ε*_405 nm_ = 11.86 mmol cm^−1^) indicating an esterase activity. The activity of all tested enzymes was calculated in Units (U). One U is defined as the amount of enzyme that is needed to catalyze the conversion of 1 μmol of substrate per minute under the given conditions.

### 2.5. Enzymatic Hydrolysis

Films (10 × 20 × 0.5 mm^3^, ~110 mg) of polymers were prepared using a heat press at 10 T and 80 °C. Polymer samples (films or powders) were placed in sealed vials containing potassium phosphate buffer (0.2 M) and incubated at the respective pH and temperature optima of the enzymes (*Pseudomonas cepacia* lipase: 37 °C, pH 7.4; *Thermobifida cellulosilytica* cutinase (Thc_Cut1):, 50 °C, pH 7.0) on a rotary shaker at 100 rpm for time intervals as indicated below. The final enzyme amounts used were 0.01 g enzyme per gram polymer. The enzyme solution was replaced every 24 h in case of the film samples. Every measurement was repeated three times. As a control, all experiments were repeated under the same conditions in the absence of the enzymes.

For analysis of % mass loss, DSC, and SEM, polymer films were removed from solution, washed thoroughly with distilled water and dried under reduced pressure until a constant weight was obtained.

For analysis of degradation products from polymer powders, the enzyme solutions were centrifuged for 15 min at 25 °C and 4000 rpm to remove powders. One mL of the supernatant was used in LC-RI analysis for detection of water-soluble release products. The pellets and remaining supernatants were used for SEC analysis whereby the hydrolysed polymers were freeze dried, suspended in THF (250 ppm BHT as inhibitor) and filtered (0.45 μm PTFE filters). The supernatants were precipitated with a modified version of Carrez precipitation [35,36]. The supernatants were acidified with HCl to pH 4. Thereafter, Carrez reagent I (20 μL) was added to the supernatant. The mixture was mixed and incubated for 1 min at 25 °C. Subsequently, Carrez reagent II (20 μL) was added to the mixture which was mixed and incubated for a further 5 min at 25 °C. The mixtures were centrifuged for 30 min at 25 °C with 14,000 rpm. Finally, supernatants were filtered (0.45 μm nylon filters) and analysed via LC-RI.

## 3. Results and Discussion

**Figure 1:**
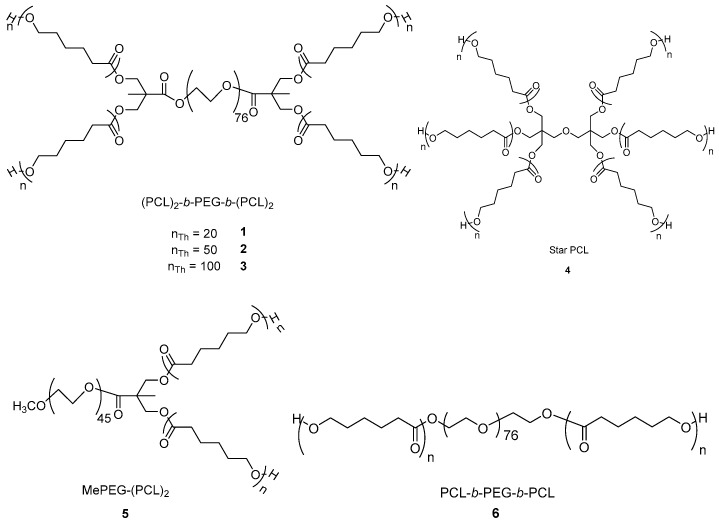
Structures of four-arm star copolymers (PCL)_2_-*b*-PEG-*b*-(PCL)_2_ **1**–**3**, six-arm star PCL with central dipentaerythritol unit **4**, Y-shaped copolymer MePEG-(PCL)_2_ **5**, and linear triblock copolymer PCL-*b*-PEG-*b*-PCL **6**.

Hydrolysis of different star-poly(*ε*-caprolactone) by two enzymes was studied. Therefore, structurally different PCLs were synthetized and characterised using previously described methods [29,30,31,32]. The model polymers included a four-arm star PCLs containing a central PEG moiety bridged by bisMPA linkages (PCL)_2_-*b*-PEG-*b*-(PCL)_2_ **1**–**3**, a six-arm star PCL containing a central dipentaerythritol unit **4**, a Y-shaped MePEG-(PCL)_2_ **5**, and a linear PCL-*b*-PEG-*b*-PCL **6** (Figure 1). The % PEG content of the copolymers were determined by the ratio of *M*_n_ of PEG over *M*_n_ of polymer (Table 1).

Star 1, Y-shaped **5**, and Linear **6** copolymers were synthesised with a PEG content of 27–30% to determine the effect of polymer architecture on enzymatic degradation rate. Star copolymers **1**–**3** containing the same central PEG moiety were synthesised with a $\bar{\mathrm{DP}}$ of 20, 50, and 100 caprolactone (*ε*-CL) units per arm, respectively, to determine the effect of PCL arm length and PEG content on enzymatic degradation rate. Star PCL **4** was synthesised without PEG moiety, to determine the effect of PEG incorporation into a star architecture on enzymatic degradation. Due to the crystalline nature of PCL homopolymers, a polymer with a relatively high molecular weight of 6.87 × 10^−4^ g mol^−1^ was synthesised to ensure it was film-forming for enzymatic degradation studies.

A large disparity can be seen in Table 1 between the theoretical *M*_n_ and that determined by SEC for star polymers 1–4 due to a linear polystyrene standard used for calibration. However, a better correlation is seen between the theoretical *M*_n_ and the *M*_n_ determined by NMR.

**Figure 2:**
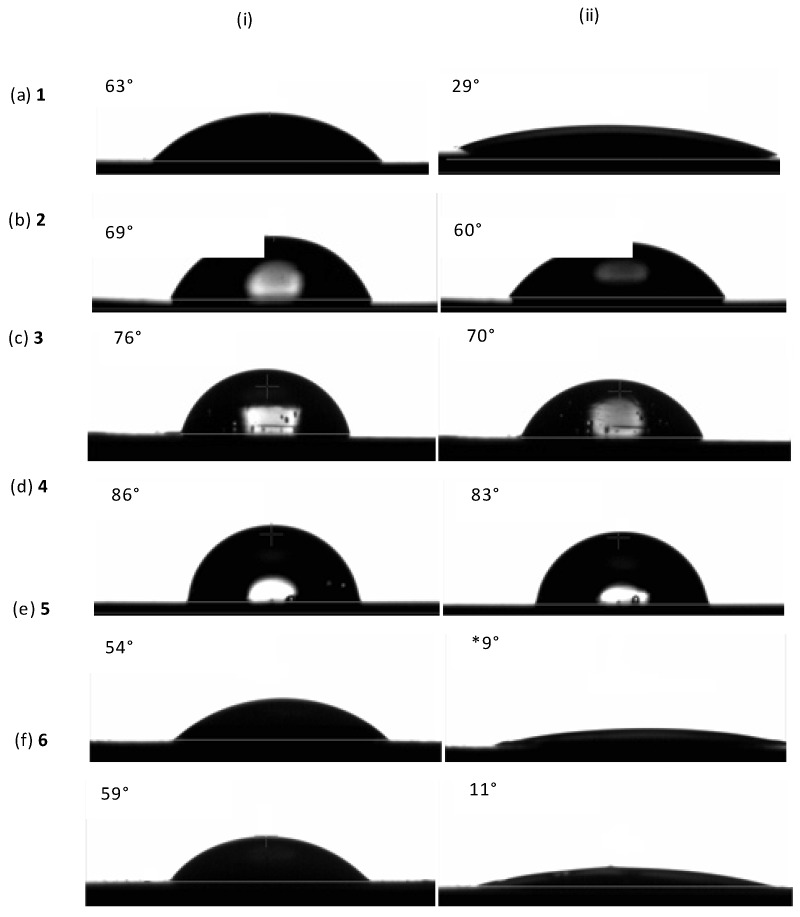
Contact Angle measurements for star copolymers **1**–**3**, star PCL polymer **4**, Y-shaped copolymer **5**, and linear copolymer **6**, for (**i**) 0 s, (**ii**) 30 s, and (ii*) 8 s.

The hydrophilic nature of copolymer films **1**–**3** and **5**–**6** was determined via contact angle analysis and compared to that of star PCL homopolymer **4** (Figure 2). It can be seen that star PCL is very hydrophobic, showing the largest initial contact angle of 86° with negligible decrease after 30 s to 83° (Figure 2d). On the other hand, the initial contact angle is seen to increase in star copolymers **1**–**3** from 63° to 76° as the %PEG^NMR^ content decreases from 26% to 7%, Figure 2a–c.

**Figure 3:**
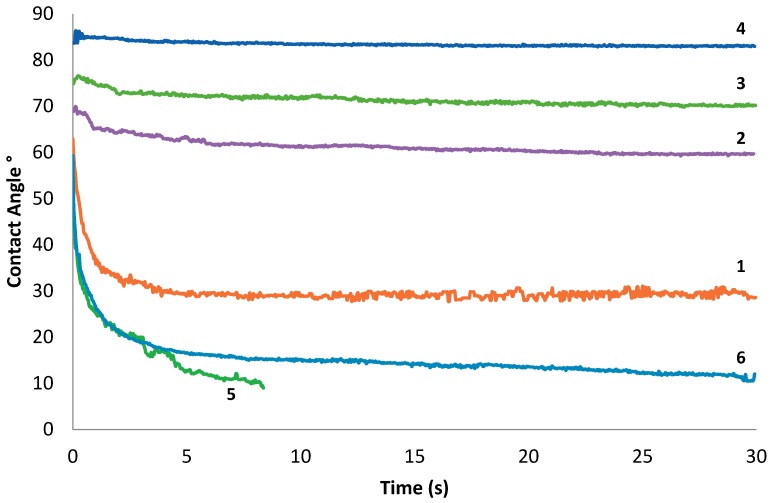
Contact angle measurements of different PCL films including star copolymers **1**–**3**, a star PCL homopolymer **4**, a Y-shaped copolymer **5**, and a linear copolymer **6** over 30 s to determine surface wettability.

The change in contact angle as a function of time for star copolymers **1**–**3**, star PCL homopolymer **4**, Y-shaped copolymer **5**, and linear copolymer **6** is presented as a graph in Figure 3. Minimal changes in contact angle from 85° to 83° over 30 s are seen for star PCL **4** and star copolymers **2** and **3** containing 0%, 13%, and 7% PEG^NMR^ content, respectively. However, star copolymer **1**, with 23% PEG^NMR^ content showed a significant decrease in contact angle from 63° to 32° within the first few seconds. Furthermore, Y-shaped copolymer **5** and linear copolymer **6** with a greater PEG^NMR^ content of 30% and 27%, respectively, show a greater decrease in contact angle from 54–63° to 21° within the first few seconds.

The lowest initial contact angle at 54° as well as the fastest decrease in contact angle to 8° after 9 s is seen for Y-shaped copolymer **5**. This can be explained with the presence of less shielded pendant MePEG chains in the Y-shaped structure, in comparison to linear copolymer **6** and four-arm star copolymers **1**–**3**. Furthermore, a clear trend of the decrease in the rate of wetting can be seen from 1–**4**, with the decrease in the %PEG^NMR^ content from 23% to 0%.

### 3.1. Enzymatic Degradation Using Lipase

**Figure 4:**
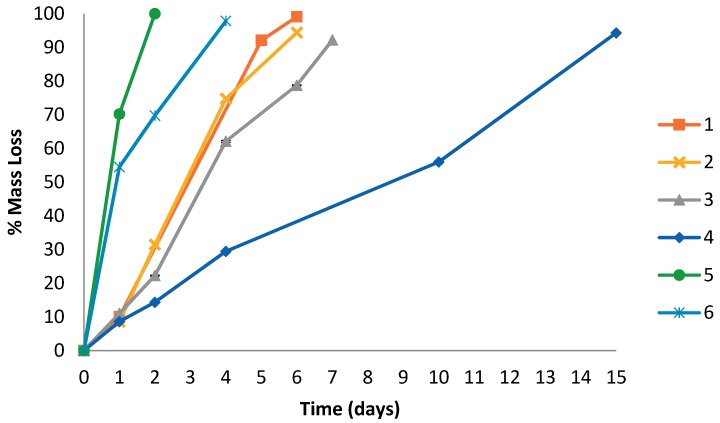
% Mass loss of different PCL films including star copolymers **1**–**3**, a star PCL homopolymer **4**, a Y-shaped copolymer **5**, and a linear copolymer **6** over time during enzymatic degradation using lipase.

The enzymatic degradation of polymer films **1**–**6** was investigated using *Pseudomonas cepacia* lipase in buffer solution. Figure 4 depicts a graph of mass loss expressed as a percentage of the initial mass as a function of enzymatic degradation time. It must be noted that the mass loss was calculated from recovered solids after degradation thus, once cleaved from the PCL arms, the water-soluble PEG moiety will remain intact in the buffer solution. A small amount of autohydrolysis was observed for all polymers with blank tests in buffer solution and in the absence of enzyme.

Star PCL **4** degrades at a significantly slower rate (>90% mass loss in 15 days) than that of the PEG containing polymers **1**–**3** and **5**–**6** (>90% mass loss in within 6 days). Moreover, during enzymatic degradation, PEG containing polymer films **1**–**3** and **5**–**6** experienced a higher degree of swelling resulting in fragmentation, than star PCL **4** which remained intact. The increased surface area due to fragmentation accelerates degradation as there is an increased surface available for the enzyme to degrade the polymer.

Y-shaped **5** and linear **6** copolymers degraded at a higher rate (>90% mass loss within 4 days) than copolymers **1**–**3** with a star architecture (>90% mass loss within 7 days). Furthermore, copolymers **1**, **5**, and **6** containing similar PEG content (27–30%) but differing architecture, all degraded at different rates. The fastest degradation (100% mass loss in 2 days) was seen in Y-shaped copolymer **5** containing a pendant MePEG unit and two PCL arms with an average degree of polymerisation ($\bar{\mathrm{DP}}$) of 22 *ε*-CL units. The second fastest degradation (>90% mass loss in 4 days) was seen with linear copolymer 6 containing a central PEG unit (*M*_n_ = 3350 g mol^−1^) and two PCL arms with a $\bar{\mathrm{DP}}$ of 40 ε-CL units. The slowest degradation of the three architectures (>90% mass loss in 5 days) was seen in star copolymer 1 containing a central PEG unit (*M*_n_ = 0.34 × 10^−4^ g mol^−1^) and four PCL arms with a $\bar{\mathrm{DP}}$ of 19 *ε*-CL units. Moreover, the fastest degradation rate seen with Y-shaped copolymer **5** could be partly attributed to a lower *M*_n_ (0.70 × 10^−4^ g mol^−1^) than star 1 (1.20 × 10^−4^ g mol^−1^) and linear **6** (1.25 × 10^−4^ g mol^−1^). Furthermore, the presence of a pendant MePEG unit in Y-shaped polymer **5** greatly increased surface hydrophilicity and wettability (Figure 2 and Figure 3), leading to increased fragmentation and mass loss.

The degradation rate of star copolymers **1**–**3** decreases as the PEG content decreases from 28% to 14% to 6%, and molecular weight increases from 1.20 × 10^−4^ g mol^−1^ to 2.71 × 10^−4^ g mol^−1^ to 5.54 × 10^−4^ g mol^−1^, respectively. This indicates that the enzymatic degradation rate using lipase is increased by increasing the hydrophilic PEG content in the copolymers. Additionally, an increase in molecular weight of the star copolymers causes a decrease in enzymatic degradation rate. This could be attributed to increased chain entanglement of star polymers as longer arms decrease the mobility and access of the enzyme between polymer chains.

The degree of crystallinity (%*χ*_c_) was calculated using Equation (1) where Δ*H*c is the measured enthalpy of crystallisation determined by DSC. Δ*H*c*_PCL_ and Δ*H*c*_PEG_ are the standard enthalpies of crystallisation for completely crystalline PCL (139.5 J g^−1^) and PEG (196.8 J g^−1^) [37].

(1) $$\%Xc=\frac{\Delta Hc}{\%\mathrm{PCL}(\Delta Hc*\mathrm{PCL})+\%\mathrm{PEG}(\Delta Hc*\mathrm{PEG})}$$

The thermal properties of polymers **1**–**6** were measured before and after enzymatic degradation using lipase at several time intervals, to determine the effect of crystallinity and thermal properties on rate of degradation (Table 2). There is a notable trend in the crystallinity of polymers before enzymatic degradation using lipase (day 0); as the PEG content of the polymer increases, the %*χ*_c_ decreases. This can be seen when the PEG content increases from 0%, 6%, 14% to 28%, the corresponding %*χ*_c_ decreases from 73%, 33%, 16% to 12% in samples 4, 3, 2, and 1 respectively.

For polymers **1**–**4** and **6**, there is a significant increase in %*χ*_c_ after 1 or 2 days of enzymatic hydrolysis using lipase and this can be explained by the enzyme firstly hydrolysing amorphous regions of the polymer film creating a more crystalline material [37]. Furthermore, the removal of the water-soluble PEG moiety due to cleavage of the PCL arms will increase crystallinity in addition to the formation of shorter PCL fragments leading to less chain entanglement and therefore a higher degree of crystallinity. The exception of this phenomenon is Y-shaped copolymer **5** whereby the %*χ*_c_ decreased from 32% at day 0 to 16% at day 1. This could be explained by the very fast rate of enzymatic degradation (70% mass loss at day 1) and an initial increase in %*χ*_c_ is expected to have occurred at a shorter time interval. A subsequent decrease in %*χ*_c_ is seen for all polymers after 3 or 4 days of enzymatic degradation as the main bulk of material is degraded.

The melting temperature (*T*_m_) for polymers **1**–**6** generally remained unchanged during enzymatic degradation, which is consistent with previously reported data [22]. However, the crystallisation temperature (*T*_c_) generally showed a slight increase, for example, from 28 to 34 °C after 5 days of enzymatic degradation, due to degradation of amorphous areas and overall increase in %*χ*_c_.

**Figure 5:**
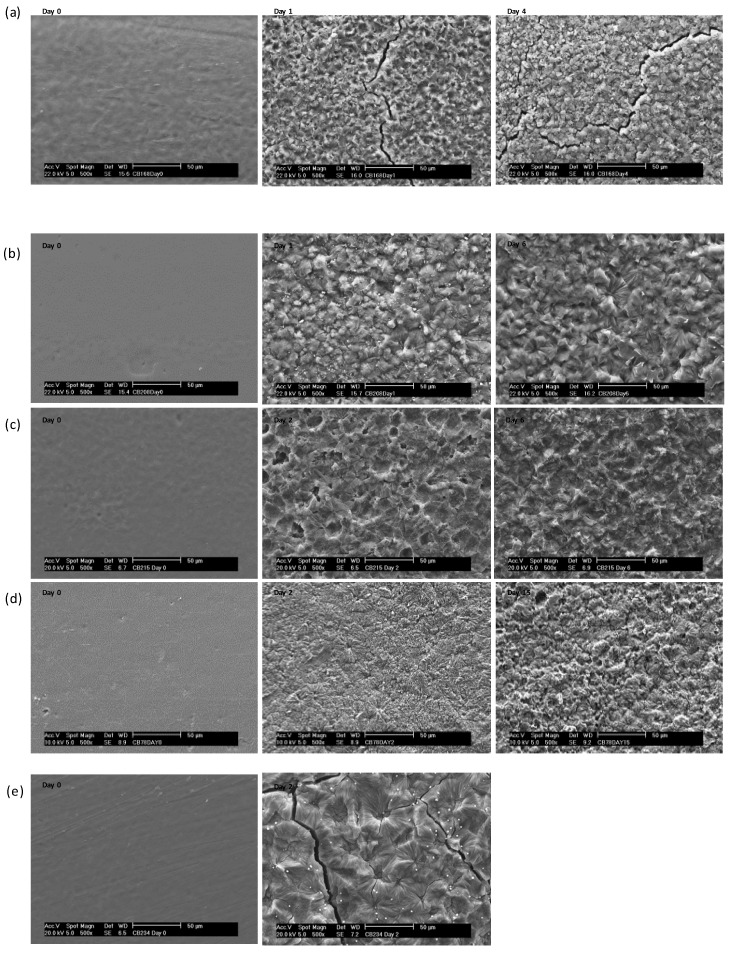
SEM micrographs of polymer films before and after enzymatic degradation using lipase on selected days (**a**) **1** at day 0, 1, and 4, (**b**) **2** at day 0, 1, and 6, (**c**) **3** at day 0, 2, and 6, (**d**) **4** at day 0, 2, and 15, and (**e**) **6** at day 0 and 2.

The surface morphology of the polymer films was analysed using SEM before and after enzymatic degradation using lipase. Figure 5 shows the SEM micrographs for polymer films **1**–**4** and **6** before degradation (day 0) and at various days throughout the degradation period. It must be noted that an SEM micrograph is not shown for polymer **5** as the film disintegrated into a fine powder after the addition of buffer solution and no film surface remained intact for analysis. Significant erosion and surface pitting over the whole surface of the film can be seen after only 1–2 days of enzymatic degradation for all polymers **1**–**4** and **6**. Furthermore, crystalline spherulites can be seen after 1–2 days of enzymatic degradation on the film surface suggesting a general increase in crystallinity. This can be explained by enzymatic degradation primarily occurring in amorphous regions of the polymer film causing an initial increase in %*χ*_c_ within the first two days, followed by a decrease in %*χ*_c_ as subsequently the crystalline areas are degraded. This is supported by the observed general increase in %*χ*_c_ for polymers **1**–**4** and **6** after 1–2 days of enzymatic degradation, determined by DSC (Table 2).

### 3.2. Enzymatic Degradation Using Cutinase

**Figure 6:**
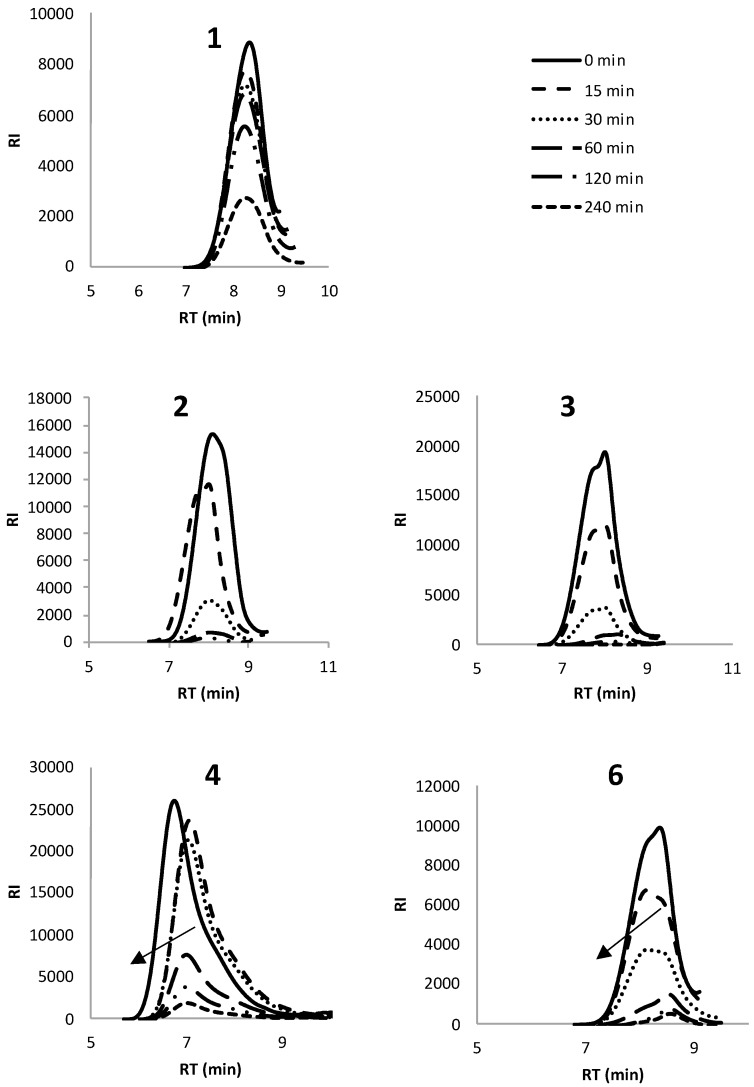
SEC chromatograms for star copolymers **1**–**3**, star PCL **4** and linear copolymer **6** after 240 min of enzymatic degradation using cutinase.

Based on the above promising results on hydrolysis of the polymers with the commercial lipase from *Pseudomonas cepacia*, in a next step, we investigated hydrolysis of the polymers more in detail by using a highly purified cutinase from the typical compost organism *Thermobifida cellulosilytica.* On the one hand, degradation of PCL polymers using cutinases has not yet been systematically investigated despite being capable to degrade a variety of synthetic polyesters [26,27,28]. On the other hand, utilization of a highly purified recombinant enzyme will allow attribution of the effect to this single protein and avoid potential impacts of minor enzyme impurities in commercial preparations. Polymers 1–6 were incubated with the *Thermobifida cellulosilytica* cutinase enzyme (Thc_Cut1) for 15, 30, 60, 120 and 240 min. Enzymatic degradation was monitored using SEC to determine the reduction in molecular weight and change in dispersity (*Ð*). As a control, enzymatic degradation tests were carried out on the synthetic precursors, PEG and MePEG using cutinase enzyme for 8 days, which showed negligible reduction in molecular weight (Figure 6, Table 3). This confirms the PEG and MePEG moieties do not degrade and remain intact throughout the enzymatic degradation tests.

Figure 6 shows a significant decrease in intensity of RI detection after 240 min of enzymatic degradation of polymers **1**–**4** and **6** by cutinase. It was not possible to monitor the rate of degradation using SEC for Y-shaped copolymer **5** due to its low *M*_n_ of 0.70 × 10^−4^ g mol^−1^ (overlapping with solvent signals) and rapid rate of degradation. This rapid rate of degradation was supported by the degradation studies with lipase, as 5 underwent the highest mass loss in the shortest time (100% in 2 days), Figure 4. The SEC chromatograms for polymers **1**–**4** and **6** show a shift to a lower molecular weight throughout the 240 min degradation period, indicating a reduction in molecular weight through the cleavage of ester bonds using cutinase enzyme.

**Figure 7:**
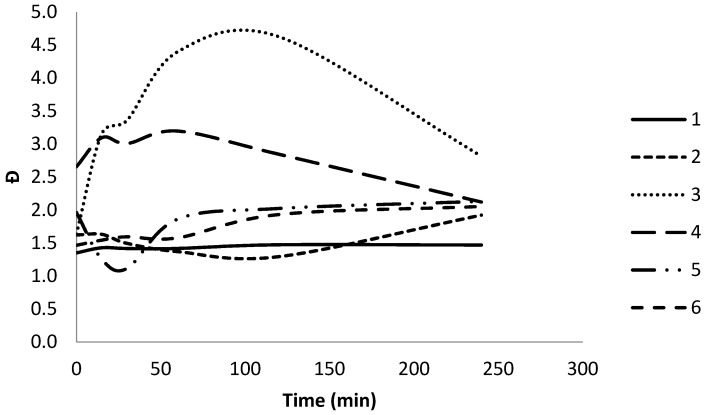
Changes in dispersity (Đ) for polymers **1**–**6** over 240 min enzymatic degradation using cutinase.

The changes in Đ for polymers **1**–**6** after 240 min of enzymatic degradation using cutinase are shown in Figure 7. It can be seen that star copolymer **3** and star PCL homopolymer **4** exhibit an initial increase in *Đ* from 1.6 to 3.2 and 2.7 to 3.1 after the first 15 min, followed by a decrease from 4.6 to 2.8 and 2.8 to 2.1 from 120 min to 240 min of degradation using cutinase, respectively. Star polymers **3** and 4 have the lowest %PEG content of 6% and 0%, respectively, indicating the cleavage of star PCL arms during enzymatic degradation increases the *Đ*. The observed *Đ* decreases due to the decreasing M_n_ of the cleaved PCL chains in the latter stages of enzymatic degradation. Copolymers **1**, **2**, and **6** containing a higher %PEG content of 14–28% showed minimal changes in *Đ* from 1.4 to 1.5, 1.6 to 1.9, and 1.5 to 2.1, respectively, in comparison to polymers **3** and **4**, throughout the 240 min of enzymatic degradation.

**Figure 8:**
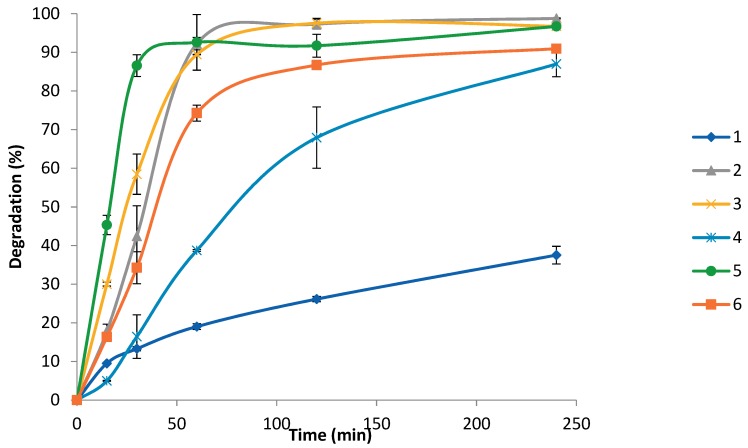
% Degradation for polymers **1**–**6** determined by release of linear and cyclic CL moieties over 250 min of enzymatic degradation using cutinase.

Furthermore, polymers **1**–**6** were analysed by LC after enzymatic degradation using cutinase, showing both 6-hydroxyhexanoic acid and *ε*-CL moieties as released degradation products. Figure 8 shows the % degradation of polymers **1**–**6** determined by the concentration of linear and cyclic *ε*-CL detected during 240 min of enzymatic degradation using cutinase. Interestingly, four-arm star copolymers 2 and 3 with 6–14% PEG content showed rapid degradation of 89–92%, during the first 60 min whereas four-arm star copolymer 1, containing the highest PEG content of 28%, showed a very slow rate of degradation of 19% after 60 min and 38% after 240 min. This suggests a decrease in %PEG content from 28% to 6% in star copolymers **1**–**3** respectively, increases the rate of enzymatic degradation using cutinase.

Y-shaped copolymer **5** and linear copolymer **6** exhibited a similar rate of degradation, and at a significantly faster rate of 74–93% after 60 min compared to four-arm star 1 with 19% after 60 min, despite all containing a similar %PEG content of 27–29%. This shows that in comparison to the four-arm star copolymer morphology of 1, the linear copolymer morphology of **6** exhibits 55% greater degradation after 60 min and the Y-shaped copolymer morphology of **5** exhibits 74% greater degradation. The relatively low rate of degradation observed for the four-arm star structure could be due to increased shielding of the central hydrophilic PEG moiety by the four hydrophobic PCL arms, therefore restricting the enzymes mobility to penetrate and degrade the bulk of the polymer. Furthermore, the pendant hydrophilic PEG moiety in Y-shaped copolymer **5** greatly increases the hydrophilicity of the polymer surface, therefore increasing the enzyme mobility and access to the polymer surface.

## 4. Conclusions

Biodegradable four-arm star PCL copolymers (PCL)_2_-PEG-(PCL)_2_ 1–3 containing a central PEG moiety were prepared and degraded using *Pseudomonas cepacia* lipase and *Thermobifida cellulosilytica* cutinase (Thc_Cut1) enzymes. As a comparison, six-arm star PCL **4**, Y-shaped copolymer MePEG-(PCL)_2_ **5**, and linear copolymer (PCL)-PEG-(PCL) **6** were prepared and degraded in a similar manner.

Contact angle analysis clearly shows an increase in hydrophilicity and wettability of the polymer films with an increase in PEG content. Increased swelling and fragmentation were seen in all PEG-containing copolymers, leading to a faster mass loss during enzymatic degradation using lipase. On the other hand, enzymatic degradation rate using cutinase generally decreased with an increase in PEG content, suggesting the PEG moiety slows down PCL degradation in copolymers. It is important to note that only PCL underwent enzymatic degradation and the water-soluble PEG moiety remained intact in the buffer solution after cleavage from PCL arms.

DSC and SEM analyses showed that %*χ*_c_ generally increased within the first two days of enzymatic degradation using lipase, as amorphous regions were primarily degraded. This was followed by a decrease in %*χ*_c_ as the bulk of material was degraded.

SEC analyses of polymers degraded using cutinase showed a significant decrease in molecular weight and general increase in dispersity after 6 h. Furthermore, HPLC analyses showed both cyclic and linear *ε*-CL degradation release products, with a maximum amount generally detected after 6 h of enzymatic degradation.

Interestingly, Y-shaped copolymer **5** exhibited the fastest rate of enzymatic degradation using both cutinase and lipase enzymes. This can be attributed to **5** containing the highest PEG content of 29%, presence of a pendant hydrophilic MePEG moiety, and comparatively low molecular weight. The slowest rate of enzymatic degradation determined by mass loss using lipase was seen with six-arm star PCL **4**. This is attributed to the high hydrophobicity and relatively high molecular weight of this homopolymer.

## Figures and Tables

**Table 1:** Shape, molecular weights, dispersities, and % PEG content for polymers **1**–**6.**

Sample	Shape	^a^ n_Th_	^b^ n_NMR_	^c^ *M*_n_^Th^	^d^ *M*_n_^NMR^	^e^ *M*_n_^GPC^	*Ð*	^f^ %PEG
				(×10^−4^ g mol^−1^)				
**1**	Star	20	19	1.28	1.20	0.96	1.47	28
**2**	Star	50	52	2.62	2.71	0.96	1.75	14
**3**	Star	100	114	4.93	5.54	1.35	1.78	6
**4**	Star	100	67	6.87	4.61	2.80	2.20	0
**5**	Y-shaped	20	22	0.67	0.70	0.68	1.36	30
**6**	Linear	40	40	1.25	1.25	0.89	1.49	27

^a^ Theoretical number of *ε*-CL units on each arm determined by feed ratio. ^b^ Average number of *ε*-CL units on each arm determined by nuclear magnetic resonance (NMR). ^c^ Theoretical molecular weight of polymer determined by feed ratio. ^d^ Molecular weight of polymer determined by NMR. ^e^ Molecular weight of polymer determined by gel permeation chromatography. ^f^ % PEG in polymer as a fraction of total weight of polymer.

**Table 2:** Thermal properties of polymer films **1**–**6** before and throughout enzymatic degradation with lipase; heat of fusion (Δ*H*_m_), degree of crystallinity (%*χ*_c_), melting temperature (*T*_m_), crystallisation temperature (*T*_c_) at cooling at 10 °C min^−1^.

Sample	PEG	Time	Mass loss	Δ*H*_m_	Δ*H*_c_	*χ*_c_	*T*_m_	*T*_c_
	%	day	%	J g^−1^		%	°C	
1	28	0	-	26	18	12	54	28
		1	10.0	64	44	29	55	36
		4	47.3	43	40	26	50	34
		5	92.1	41	41	26	51	34
		6	99.1	-	-	-	-	-
2	14	0	-	36	24	16	57	34
		1	8.6	88	60	41	56	38
		2	31.5	58	60	41	52	37
		4	74.8	72	71	48	52	38
		6	94.4	51	50	34	52	38
3	6	0	-	68	47	33	59	33
		1	11.0	66	70	46	57	34
		2	22.3	61	61	43	59	34
		4	62.1	48	53	34	57	34
		6	78.8	71	73	50	55	36
4	0	0	-	90	101	73	58	31
		1	8.6	36	41	29	58	31
		2	14.3	42	48	34	57	31
		4	29.5	10	11	8	57	32
		15	94.3	4	5	3	56	31
5	29	0	-	51	50	32	50	27
		1	70.2	37	25	16	57	30
		2	100	-	-	-	-	-
6	27	0	-	40	40	26	54	29
		2	69.8	52	51	33	54	34
		4	97.8	-	-	-	-	-

**Table 3:** Changes in molecular weight for polymers **1**–**6** after 240 min of enzyme degradation using cutinase determined by SEC analysis. *M*_w_**:** Weight-average molecular weight *M*_n_**:** Number-average molecular weight *Đ***:** Dispersity.

Polymer	Enzymatic degradation	*M*_n_	*M*_w_	*Đ*
	(min)	(×10^4^ g mol^−1^)		
**1**	0	0.57	0.77	1.4
	15	0.62	0.65	1.4
	30	0.55	0.77	1.4
	60	0.54	0.77	1.4
	120	0.52	0.77	1.5
	240	0.51	0.75	1.5
**2**	0	0.64	1.03	1.6
	15	0.83	1.35	1.6
	30	0.75	1.12	1.5
	60	0.75	0.80	1.4
	120	0.93	1.29	1.3
	240	0.91	1.72	1.9
**3**	0	0.98	1.61	1.6
	15	0.39	1.24	3.2
	30	0.34	1.17	3.4
	60	0.13	0.55	4.4
	120	0.07	0.33	4.6
	240	0.07	0.22	2.8
**4**	0	2.30	6.11	2.7
	15	1.26	3.89	3.1
	30	1.29	3.87	3.0
	60	1.20	4.85	3.2
	120	0.93	3.80	2.8
	240	1.92	4.06	2.1
**5**	0	0.17	0.33	2.0
	15	0.23	0.29	1.3
	30	0.69	0.79	1.1
	60	0.54	1.00	1.9
	120	0.39	0.79	2.0
	240	0.35	0.75	2.1
**6**	0	0.58	0.86	1.5
	15	0.54	0.83	1.5
	30	0.51	0.81	1.6
	60	0.38	0.60	1.6
	120	0.21	0.57	1.9
	240	0.20	0.40	2.1
